# New and Emerging Targeted Therapies for Pediatric Acute Myeloid Leukemia (AML)

**DOI:** 10.3390/children7020012

**Published:** 2020-02-10

**Authors:** Jing Chen, Chana L. Glasser

**Affiliations:** 1 Division of Pediatric Hematology/Oncology, Hackensack University Medical Center, Hackensack, NJ 07601, USA; 2 Division of Pediatric Hematology/Oncology, NYU Winthrop Hospital, Mineola, NY 11501, USA

**Keywords:** acute myeloid leukemia, antibody drug conjugate

## Abstract

The relapse rate for children with acute myeloid leukemia (AML) remains high despite advancements in risk classification, multi-agent chemotherapy intensification, stem cell transplantation, and supportive care guidelines. Prognosis for this subgroup of children with relapsed/refractory AML remains poor. It is well known that the ceiling of chemotherapy intensification has been reached, limited by acute and chronic toxicity, necessitating alternative treatment approaches. In the last several years, our improved understanding of disease biology and critical molecular pathways in AML has yielded a variety of new drugs to target these specific pathways. This review provides a summary of antibody drug conjugates (ADCs), small molecule inhibitors, and tyrosine kinase inhibitors with an emphasis on those that are currently under clinical evaluation or soon to open in early phase trials for children with relapsed/refractory AML.

## 1. Introduction

Acute myeloid leukemia (AML), a hematopoietic stem cell disorder characterized by clonal expansion of abnormally differentiated myeloid lineage blasts, accounts for 20% of childhood leukemia [1]. AML continues to pose a significant therapeutic challenge due to disease heterogeneity, high relapse rate, and therapy toxicity [2]. As compared to a 90% overall survival (OS) in childhood acute lymphoblastic leukemia (ALL), the OS approaches only 69–75% in childhood AML at best [2,3,4]. Despite significant advancements in risk classification and frontline therapeutic approaches, about 50% of children with AML relapse and prognosis for these children remains poor [3]. Recently published data generated from the Therapeutically Applicable Research to Generate Effective Treatments (TARGET) AML initiative, a collaborative Children’s Oncology Group (COG)–National Cancer Institute (NCI) project to characterize the mutational, transcriptional, and epigenetic landscape of childhood AML, have significantly broadened our understanding of the biology of AML in children and how it differs from adults. Large-scale retrospective whole genome, DNA, and RNA sequencing as well as methylation profiling has revealed novel fusion genes, focal deletions, and recurrent mutations unique to pediatrics, some of which are associated with particularly poor prognoses. It has become clear that pediatric AML is a collection of molecularly diverse diseases with similar phenotype which has led to a refinement of our molecular risk classification and increased efforts toward personalized targeted therapy approaches [5]. It is evident that the ceiling of chemotherapy intensification has been reached, limited by infectious and cardiac toxicity, urgently necessitating novel therapeutic strategies. Alternate approaches to cytotoxic chemotherapy including epigenetic therapy, immunotherapy, antibody drug conjugates (ADCs), and small molecule inhibitor therapies are revolutionizing cancer care. In this review, we focus on the role of ADCs, small molecule inhibitors, and tyrosine kinase inhibitors in the treatment of childhood AML, discussing existing and emerging drugs that hold great promise in improving outcomes.

## 2. Antibody Drug Conjugates

**Figure 1:**
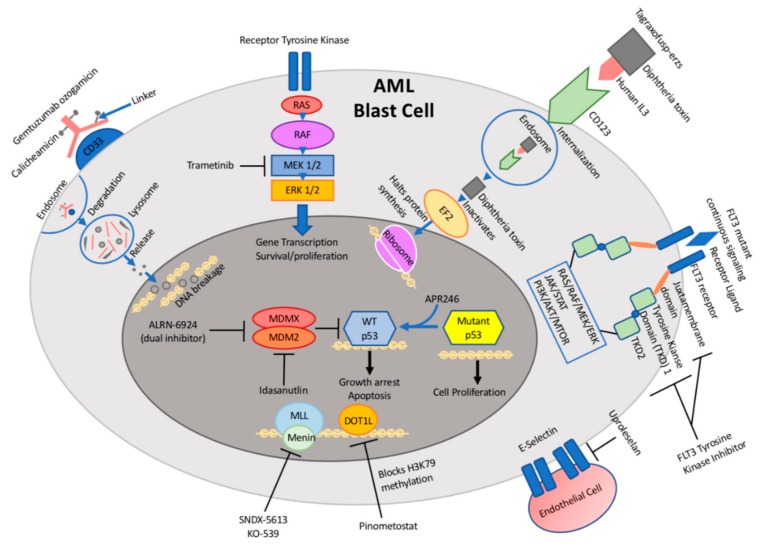
Schematic illustration of targetable pathways and drug mechanisms in pediatric acute myeloid leukemia (AML).

Antibody-based therapies have emerged as an effective and well-tolerated approach to cancer care, whereby attaching a cytotoxic drug to an antibody can lead to increased dose intensity with reduced toxicity [6] (Figure 1, Table 1). The challenge in AML has been to identify targetable cell surface proteins that are expressed in AML blasts but not in normal hematopoietic cells.

### 2.1. Targeting CD33

The cell surface antigen, CD33, is present in more than 80% of patients with AML but is absent from hematopoietic stem cells, rendering it an ideal immunoconjugate target [2]. From early on, agents that were pursued as therapeutics included unconjugated antibodies, radioimmunoconjugates, ADCs, and immunotoxins; however, success with CD33-targeted therapeutics has been limited with several agents failing at various stages of clinical development [7]. For example, Lintuzumab (Seattle Genetics, HuM195, SGN-33), an unconjugated humanized murine monoclonal antibody, showed great promise in vitro eliciting antibody-dependent cellular cytotoxicity (ADCC) and phagocytosis leading to AML cell killing. However, this activity did not translate successfully to the clinic in adult clinical trials, resulting in termination of drug development [7,8,9]. The newer generation, vadastuximab talirine (Seattle Genetics, SGN-CD33A), a CD33 directed antibody conjugated to pyrrolobenzodiazepine (PBD) dimers, has shown more promise in relapsed/refractory (r/r) adult patients as monotherapy and in combination with a hypomethylating agent, but concerns over hepatic and hematopoietic toxicity derailed drug development [7,10,11]. 

The most promising data for targeting CD33 in both children and adults have come from studies with Gemtuzumab ozogamicin (Mylotarg, GO, Pfizer, New York, USA), a humanized IgG4 anti-CD33 antibody conjugated to calicheamicin, a DNA-cleaving cytotoxic agent, leading to its FDA approval in 2000. A subsequent randomized controlled trial in adults, Southwest Oncology Group (SWOG) S0106, failed to meet its primary end points of improved remission induction and safety, resulting in withdrawal of approval [2,12]. However, the results of this study were controversial and other concurrent adult studies did show improved survival with the addition of GO [13,14]. COG demonstrated feasibility and safety in adding 2 doses of GO to standard intensive chemotherapy for children with previously untreated AML in their pilot study AAML03P1 [15]. In the subsequent randomized controlled trial, AAML0531, newly diagnosed pediatric AML patients were randomized to receive the standard upfront chemotherapy backbone with or without 2 doses of GO at 3 mg/m^2^/dose. This landmark study showed that 3-year event free survival (EFS) was significantly improved with the addition of GO (53% vs. 46.9%, *p* = 0.04) by a significant reduction in 3-year relapse risk (RR) (32.8% vs. 41.3%, *p* = 0.006), particularly in the low and intermediate risk groups. Furthermore, the study did not demonstrate an increased risk of sinusoidal obstructive syndrome (SOS) in the GO group as had been the case in early studies using higher doses of GO [2]. Based on the results of these studies, GO again earned Food and Drug Administration (FDA) approval in 2017 for the treatment of newly diagnosed CD33-positive AML in r/r adults and children ≥2 years of age [16]. This success story has led to the incorporation of GO into the backbone of the upcoming COG randomized controlled clinical trial, AAML1831, comparing CPX-351, a liposomal preparation of cytarabine and daunorubicin versus standard cytarabine and daunorubicin, expected to open for enrollment in the first quarter of 2020.

### 2.2. Targeting Mesothelin

Mesothelin is a cell-surface tumor differentiation antigen expressed on mesothelial cells of serosal lining. It has been associated with malignant transformation, cellular proliferation, and tumor aggressiveness in a variety of solid tumors, including lung, pancreatic, and ovarian origin. Mesothelin was recognized as an attractive candidate for targeted cancer therapy due to its limited expression in normal tissue and high expression in cancer tissue [17,18]. Anetumab ravtensine (AR) (Bayer, Leverkusen, Germany) is an ADC that contains a human anti-mesothelin antibody conjugated to the maytansinoid tubulin inhibitor DM4 via a reducible disulfide linker [19]. Preclinical studies have shown potent antitumor activity in adult solid tumor models [19,20], which has led to the development of a number of Phase I and II clinical trials for adults with aggressive mesothelin-expressing solid tumors alone and in combination therapy [17]. Mesothelin was also shown to be expressed in pediatric AML cells [21]. Building on this finding, as part of the NCI/TARGET AML initiative, transcriptome sequencing (RNA-seq) was performed on AML cell lines which demonstrated that mesothelin was one of the most highly expressed genes in ~30% of childhood AML cases, a higher prevalence than in adult AML cases (~11%). Therefore, they conducted in vitro and in vivo studies with mesothelin-overexpressing AML cell lines and xenografts, respectively, and found that treatment with AR resulted in significant mesothelin-dependent efficacy at clinically achievable doses [22,23]. Furthermore, they demonstrated in vivo synergy between mesothelin-targeted therapy and conventional chemotherapy in mesothelin+ AML xenografts [24]. Based on this promising data and emerging safety and efficacy data from adult solid tumor clinical trials, a new Phase I COG study, AAML2011, is currently in development to assess treatment with AR for patients with r/r mesothelin-expressing AML.

### 2.3. Targeting CD123

CD123, the alpha subunit of the IL-3 receptor, is overexpressed in multiple hematologic malignancies, including AML, ALL, and blastic plasmacytoid dendritic cell neoplasm (BPDCN). Because of its high expression on leukemic blasts as compared with normal hematopoietic stem cells, CD123 has emerged as an attractive candidate for molecularly targeted therapeutics [25]. Tagraxofusp-erzs (Elzonris, Stemline) and IMGN632 (immunogen) are two anti-CD123-directed immunotoxins which have been developed in recent years. Tagraxofusp-erzs is a novel biologic targeted therapy, comprised of human IL-3 coupled to a truncated diphtheria toxin payload that inhibits protein synthesis directed at the interleukin-3 receptor [26]. In December 2018, Tagraxofusp-erzs gained FDA approval for treatment of BPDCN in adult and pediatric patients ≥2 years of age. The approval was based on results of a single arm study, STML-401-0114, in which the pivotal cohort of 13 treatment-naïve BPDCN patients, treated with Tagraxofusp-erzs monotherapy, showed a 54% composite complete remission (CRc) rate and safety was established in 94 patients with myeloid neoplasms [27,28].

IMGN632 is comprised of a novel humanized anti-CD123 antibody, G4723A, linked to a unique DNA-alkylating payload of the recently developed IGN (indolinobenzodiazepine pseudodimer) class of cytotoxic compounds [25,29]. Kovtun et al. showed that IMGN632 demonstrated potent activity in all AML samples at concentrations well below levels that impacted normal bone marrow progenitors and exhibited robust antitumor activity with a wide therapeutic index in multiple AML xenografts [25]. Subsequently, Daver et al. conducted the first Phase I study of IMGN632 in adult patients with r/r AML and other CD123-positive hematologic malignancies (National Clinical Trial (NCT) 03386513), which is still recruiting. At the time of analysis, they found that 20% of AML patients (*n* = 66) and 43% (*n* = 7) of BPDCN patients had an objective response (complete remission (CR), complete remission with incomplete hematologic recovery (CRi), and partial remission (PR)) to IMGN632 [30]. These encouraging results have led to the development of a Phase Ib/II trial (NCT04086264) planned to evaluate IMGN632 as monotherapy or in combination with Venetoclax and/or Azacytidine for adults with untreated CD123+ AML ineligible for standard upfront therapy, minimal residual disease (MRD) positive, or r/r disease [31]. Through the Pediatric Acute Leukemia (PedAL) Initiative, the COG is currently developing a study for children with r/r leukemias, with a Phase I dose finding monotherapy arm for ALL and AML followed by a Phase II safety arm in combination with chemotherapy, followed by a Phase III randomization of chemotherapy ± IMGN632.

## 3. Small Molecule Inhibitors

With the discovery of multiple pathways critical to the proliferation and survival of AML cells, small molecule inhibitors have emerged as a promising therapeutic strategy in AML (Figure 1, Table 1). Small molecule inhibitors have the advantage of being able to target cell surface ligand receptors and intracellular proteins that promote the downstream signaling pathway for cell growth and metastatic disease. Targeted therapy with small molecule inhibitors, such as tyrosine kinase inhibitors (TKIs) for t(9;22) BCR-ABL gene fusion for Chronic Myeloid Leukemia (CML) or all-trans retinoic acid (ATRA) for acute promyelocytic leukemia (APML), have already proven to be successful [32]. The challenge in identifying potential targets in AML has been its heterogeneous genetic and molecular make up, with frequent evolution of the driver mutations at disease onset and progression.

### 3.1. E-Selectin Inhibitors

Binding of E-selectin adhesion molecules to AML blasts enhances the adherence of AML cells within the vascular bone marrow niche via activation of the Wnt signaling pathway. Protection within the vascular marrow niche supports leukemia cell quiescence, limits their exposure to cell-cycle dependent chemotherapy, and prolongs their survival [33,34,35]. The majority of primary AML blasts express E-selectin ligand, with increased expression in relapsed patients [35]. In addition, the higher expression of E-selectin associated with relapsed disease correlates with poor survival and high-risk disease in AML [36]. Because of its high expression and the protective role in AML cells, E-selectin has emerged as an ideal candidate for targeted therapy.

Uproleselan (Glycomimetics (GMI)-1271) is a small molecule inhibitor designed to prevent the binding of E-selectin to ligands on leukemia blasts. Preclinical work demonstrated that GMI-1271 can overcome adhesion-mediated chemotherapy resistance in vitro and reduce leukemia burden in mice engrafted with primary AML cells [35,37]. Uproleselan was granted Breakthrough Therapy Designation by the US FDA in 2017 for adults with r/r AML based on the notable result produced from a Phase II clinical trial in combination with mitoxantrone/etoposide/cytarabine (MEC) chemotherapy. This resulted in a CR/CRi for 22 out of 54 patients (41%), MRD-negative remission for 11 out of 16 evaluable patients (69%) at the recommended Phase II dose, and a median overall survival of 8.8 months. In addition, those with high expression of E-selectin ligand demonstrated greater inhibition by uproleselan and significantly longer overall survival [38]. A Phase III trial is ongoing to investigate the efficacy of uproleselan with MEC chemotherapy compared to MEC alone in adults with r/r AML (NCT03616470). The results of this trial have not yet been published. The study of E-selectin and its inhibitors in pediatric AML has been minimal; however, a recent report demonstrated that E-selectin ligand expression is also associated with poor survival in a small cohort of AML patients treated on COG AAML1031 [39]. Plans are being made for an early phase trial of chemotherapy ± uproleselan in children with r/r AML through the PedAL Initiative.

### 3.2. Targeting KMT2A-Fusion

*KMT2A* (formerly known as *MLL or mixed lineage leukemia*)-rearranged acute leukemia, involving fusions of 11q23, is a particularly aggressive leukemia affecting approximately 15–20% of childhood AML. KMT2A rearrangements are found in several groups of leukemia patients: Infantile ALL, AML, and acute megakaryoblastic leukemia (AMKL), a rare subtype of AML that occurs mainly in children with Down syndrome [40,41,42]. Outcome remains poor for KMT2A-rearranged leukemia following current conventional chemotherapy, although prognosis for AML is dependent on its fusion partner, of which over 100 fusion partners are known to date [40,41]. Clearly, there is a need for new treatment modalities for this group of patients. Two classes of small molecule inhibitors have emerged as possible therapies for KMT2A-rearranged leukemia: Disruptor of telomeric silencing 1-like (DOT1L) inhibitors and menin inhibitors.

DOT1L (disruptor of telomeric silencing 1-like) is a histone methyltransferase at H3K79 that in its normal form transcriptionally modifies chromatin of downstream target genes to help maintain the integrity of chromosomes and genes. However, the aberrant recruitment of DOT1L by a majority of KMT2A-fusion partners results in an inappropriate hypermethylation and overexpression of downstream, targeted genes that promote leukemia. The oncogenic drive of KMT2A-rearranged leukemia is dependent on the activation of DOT1L [43,44]. Therefore, the inhibition of DOT1L activation is an attractive target for new drug development. Preclinical work on DOT1L inhibitors in both cell lines and mice transformed with KMT2A-rearranged leukemia demonstrated inhibition of tumor proliferation and leukemia regression [45]. Pinometostat (Epizyme (EPZ)-5676), a DOT1L inhibitor, was one of the first histone methyltransferase inhibitors to undergo Phase I trials for both children and adults with r/r KMT2A-rearranged acute leukemia. However, single agent therapy with Pinometostat demonstrated very modest anti-leukemic activity in a cohort of 51 adults with r/r acute leukemia, a majority with KMT2A-rearrangement, resulting in 2 CR, both with t (11,19), 3 patients with resolution of leukemia cutis, and 9 patients with morphological evidence of myeloid differentiation [46]. In children with r/r, KMT2A-rearranged acute leukemia, there was no objective response as defined by protocol, although 7 out of 18 children showed evidence of transiently reduced peripheral blood or marrow blasts without meeting thresholds for objective response during therapy [47]. Although not significantly efficacious as a single agent, Pinometostat combined with azacitidine (NCT03701295) in KMT2A-rearranged r/r AML is currently being investigated in adults.

Another area of therapeutic interest has been to inhibit the binding between MLL and menin, an essential co-factor of the oncogenic component of the KMT2A complex that also acts as a histone methyltransferase to transcriptionally regulate targeted genes that are critical to leukemogenesis. Several small molecule menin inhibitors have been developed to date [48,49]. One of the earliest hallmark preclinical studies of first generation menin inhibitors, MI-463 and MI-503, demonstrated high potency inhibition of menin in KMT2A-rearranged leukemia cell lines and a substantial survival benefit in mice models harboring KMT2A-rearranged leukemia [48]. The most promising preclinical work has resulted from Vitae Pharmaceuticals (VTP)-50469 (close analog of Syndax (SNDX)-5613), an orally available, potent, and highly selective menin inhibitor which significantly inhibited proliferation of tumor growth in vitro and in vivo [44]. The robustness and consistency of these preclinical results have recently gained the approval of SNDX-5613 to enter into a Phase I/II clinical trial for r/r acute leukemia in adults, including those with KMT2A-rearranged ALL or AML, in late 2019 (NCT04065399). In children, however, VTP-50469 has only been investigated in preclinical, patient-derived xenograft (PDX) models harboring KMT2A-rearranged acute lymphoblastic leukemia. A report at the annual American Association for Cancer Research (AACR) meeting in 2018 revealed that 6 out of 7 of these PDX models demonstrated significantly longer EFS (ranging 1.1 to 109 days) compared to a non-KMT2A-rearranged PDX model following treatment with VTP-50469. Plans are being made for an early phase trial of chemotherapy ± SNDX-5613 in children with r/r acute leukemia through the PedAL Initiative [50].

Finally, another menin-KMT2A inhibitor, Kura Oncology (KO)-539, has been shown to inhibit leukemia growth and prolong survival in KMT2A-rearranged cell lines and in vivo models, earning the FDA Orphan Drug Designation [51]. This compound also recently entered a Phase I/II trial in adults with r/r AML (NCT04067336).

Targeting more than one component of the KMT2A complex has great potential for targeted treatment. Although a DOT1L inhibitor by itself has not shown promising results in Phase I trials, Dafflon et al. showed that the combination of a DOT1L inhibitor and a menin inhibitor demonstrated robust synergy in its anti-leukemic growth inhibition effect in a mouse model injected with KMT2A-AF9 leukemic cells, compared to either drug alone [44,52]. Further studies will be needed to investigate whether the combination of Pinometostat with another anti-leukemic agent can improve its efficacy.

### 3.3. MEK Inhibitors

Mitogen-activated protein kinase (MAPK) cascade is a signaling pathway crucial for the regulation of cell differentiation, proliferation, and survival. The cascade is activated by ligand binding to a receptor tyrosine kinase (RTK) to activate Ras then Raf, followed by Mitogen-activated protein kinase (MAPK, also known as MEK), finally resulting in activation of the last component of the pathway: Extracellular signal-regulated kinase (ERK). Activated ERK can then activate a variety of downstream substrates, and so the suppression of MEK/ERK activation can have profound effects on controlling cell growth [53,54,55,56]. Ras pathway mutations are highly prevalent in relapsed acute leukemia, especially in pediatric AML where it can represent up to 30% of cases, as *KRAS*, *NRAS*, fms-like tyrosine kinase 3 (*FLT3*) or *PTPN11* mutations [5]. RAS pathway mutations are particularly common with KMT2A fusions [5], core-binding factor AML [57], and normal karyotype. Although it is not formally defined as a poor prognostic marker, it also does not significantly impact overall or event-free survival. RAS pathway mutations are nevertheless associated with high risk features as stated above. Due to difficulty in targeting RAS itself, most of the effort has concentrated on inhibiting downstream effectors such as MEK [58]. The most studied of the MEK inhibitors has been trametinib, a MEK 1/2 inhibitor.

The preclinical efficacy of MEK inhibitors in reducing leukemia burden and prolonging survival in cell lines, primary patient cells, and mouse xenograft models harboring RAS-mutated acute leukemia cells has been demonstrated in a variety of studies [59,60]. Data are not as robust in childhood acute leukemia, but ex vivo drug sensitivity testing on RAS-mutated primary ALL and AML cells from children enrolled onto Leukemia Precision-based Therapy (LEAP) Consortium (NCT026770525) has demonstrated dose-response sensitivity of leukemia cells to trametinib [61]. To date, the COG has only investigated a MEK inhibitor for acute leukemia through one of its registered studies, which is an ongoing Phase II trial of trametinib in r/r juvenile myelomonocytic leukemia (JMML). The results have not yet been published. Clinically, trametinib showed an overall response rate of 20% as a single-agent therapy in a cohort of r/r NRAS/KRAS-mutated AML in adults [62]. Plans are being made for an early phase trial of chemotherapy ± trametinib in children with r/r, RAS-mutated ALL or AML through the PedAL Initiative.

### 3.4. MDM2 Antagonists

Although Tumor Protein p53 (*TP53*) mutation is an infrequent event in AML [63], the inactivation of wild-type p53 occurs in a majority of AML. Multiple mechanisms exist for the inactivation of p53. The best-studied mechanism is by an overexpression of MDM2 (murine double minute 2). MDM2, like TP53, is also expressed in a variety of human cancers. More importantly, its overexpression is reported in up to 50% of AML [63,64]. MDM2 is primarily a negative regulator of the activity of TP53, binding to the transcriptional domain of TP53 to prevent it from binding to specific DNA sequences and marking P53 for proteasomal degradation. In this way, an overexpression of MDM2 in AML allows for the amplification of the MDM2–TP53 interaction to further limit the tumor suppressor functions of wild-type TP53 [65,66,67]. In addition, the overexpression of MDM2 is also associated with shorter CR duration and event-free survival [64]. Therefore, MDM2 antagonists have emerged as a promising anti-cancer agent to block the MDM2–TP53 interaction and restore the tumor suppressor functions of TP53.

Among the first studied small-molecule MDM2 antagonists are the Nutlins [68], which bind to MDM2 in the p53-binding pocket to activate p53 signaling pathways. As early as 2004, Nutlins demonstrated cell-cycle arrest, apoptosis, and tumor growth inhibition in AML cell lines, primary AML samples, and xenograft mice models harboring wild-type p53. As expected, those harboring p53 mutations were resistant to Nutlins [68,69]. More recent preclinical studies continue to support the in vitro and in vivo anti-leukemic effects of Nutlins in wild-type p53-expressing AML cells [70]. Idasanutlin, a second-generation Nutlin, was developed to achieve more potency and less toxicity than early generation Nutlins. A Phase Ib/II trial of Idasanutlin with Cytarabine in adults with r/r AML achieved a CR rate of 25% and a CRc rate of 29% [71]. Idasanutlin is now being investigated in a Phase III trial in r/r adult AML in combination with Cytarabine compared to Cytarabine alone (NCT02545283). The results have not yet been published. Idasanutlin is also being investigated in a Phase Ib/II trial for r/r adult AML in combination with venetoclax, a B cell lymphoma-2 (BCL-2) inhibitor (NCT02670044) based on synergistic effect in preclinical studies [72]. Early clinical data have shown the combination to be safe with an anti-leukemic effect of 50% and a CR/CRi/CRp rate of 29% at the recommended Phase II dose [73]. A review on the efficacy of Idasanutlin in AML was recently published [74]. In pediatrics, Carol et al. also demonstrated high anti-leukemic activity of RG7112 (Idasanutlin) as a single agent in pediatric ALL xenografts [75]. In children with r/r AML, Aileron (ALRN)-6924 (a dual MDM2/MDMX inhibitor) in combination with Cytarabine is now being investigated for the first time through an ongoing Phase 1 trial (NCT03654716).

### 3.5. Targeting Mutant TP53

The most frequent genetic variation across all human cancers is mutation of the tumor suppressor gene TP53. It is present mainly as a missense point mutation in up to 50% of some of the most common adult cancers, including head and neck cancers, pancreatic, colorectal, and ovarian [76]. In comparison, it is an infrequent occurrence in acute leukemia. About 8% of young adults and older with AML have TP53 mutations [63,77], although it has been reported in up to 18% of adults with newly diagnosed AML [78]. It is even less frequent in children with AML [5]. Despite its infrequent occurrence, it is still associated with therapy-related myelodysplastic syndrome (MDS)/AML, relapsed AML, and poor survival compared to TP53 wild-type AML [63,78]. Mutations of TP53 lead to loss of wild-type functions, including intrinsic tumor suppressive properties such as cell apoptosis and senescence. This results in cancer cell proliferation and survival with promotion of metastatic disease and chemoresistance [63,79].

Unlike MDM2 antagonists which aim to target the overexpression of a TP53-interacting protein, targeting the mutant TP53 itself is also of therapeutic interest. The most successful trial to date in AML has been testing Aprea Therapeutics (APR)-246, a methylated, small-molecule derivative of PRIMA-1 (p53 re-activation and induction of massive apoptosis), which has been shown to bind and reactivate mutant and inactivated p53 by restoring its wild-type transcriptional activity to induce cell apoptosis [80,81]. An ongoing Phase Ib/II clinical trial investigating APR-246 combined with Azacitidine in adults with TP53-mutated MDS and oligoblastic AML (≤30% blasts) was recently presented at the 2019 American Society of Hematology annual meeting (NCT03072043). The Phase II component of the trial achieved a CR rate of 50% for AML and an overall response rate (ORR) of 88% for MDS/AML with a well-tolerated combination. Median time to response was 2.1 months and the median duration of response was 6.5 months. In addition, an isolated TP53 mutation was predictive of a higher CR rate following APR-246 therapy [82]. In April 2019, APR-246 was granted Fast Track and Orphan Drug designations for the treatment of adult MDS with TP53 mutation by the FDA. These promising results also supported the ongoing adult Phase III trial of Azacitidine ± APR-246 in TP53-mutated MDS (NCT03745716). Further work will be needed to elucidate which of the thousands of TP53 mutations may be better targeted by APR-246.

## 4. FLT3 Inhibitors

FLT3 (fms-like tyrosine kinase 3), a cytokine receptor (CD135) belonging to the receptor tyrosine kinase class III, is expressed mainly on hematopoietic cells and plays a pivotal role in myeloid and lymphoid cell proliferation and survival. *FLT3* internal tandem duplications (FLT3-ITDs), the most common class of *FLT3* gene mutations found in AML, lead to constitutive activation of the kinase, promoting cell growth, survival, and anti-apoptotic signaling. FLT3-ITDs are associated with a poor prognosis due to a high relapse rate [6]. There has been great excitement in developing targeted FLT3 kinase inhibitors over the past two decades and these agents have improved outcomes in this AML subtype (Figure 1, Table 1).

First generation FLT3 inhibitors including sorafenib and midostaurin are multi-kinase inhibitors that show activity against a host of growth factors, including FLT3, KIT, platelet-derived growth factor receptor (PDGFR), and vascular endothelial growth factor (VEGF), leading to more off-target toxicity. Sorafenib is FDA-approved for treatment of renal cell carcinoma. There have been mixed results in early phase clinical trials of Sorafenib in combination with chemotherapy for adults with de novo FLT3-ITD+ AML [83,84]. However, the COG AAML 1031 clinical trial, evaluating the addition of Sorafenib to standard chemotherapy as well as maintenance sorafenib following hematopoietic stem cell transplant (HSCT) for children with high allelic ratio FLT3-ITD+ AML showed improved CR rates as compared to historical controls following induction I (73% vs. 56%, *p* = 0.078) and following induction II (91% vs. 70%, *p* = 0.007). Furthermore, 3-year EFS was improved to 57.5% from the historical control of 34.3%, *p* = 0.007, and the RR was reduced with sorafenib treatment (18.2% vs. historical 52.5%, *p* = 0.006), although 3-year OS was not significantly different (63.9% vs. 54.1%, *p* = 0.375) [85]. Sorafenib is also being investigated in a Phase I trial in combination with Palbociclib in adolescents and adults with R/R leukemia (NCT03132454).

Midostaurin, another first-generation FLT3 inhibitor, is the first tyrosine kinase inhibitor to be FDA approved for AML therapy in the first line. Approval largely resulted from the Alliance 10603 trial, in which adults with newly diagnosed, FLT3-mutated AML, who received midostaurin in combination with standard chemotherapy had superior EFS and OS as compared to standard chemotherapy alone [86]. In children, midostaurin has been investigated as monotherapy in r/r pediatric leukemia, including KMT2A-rearranged ALL or FLT3-mutated AML (NCT0866281), which unfortunately demonstrated limited efficacy in the 22 enrolled participants (the trial terminated early due to lack of enrollment) [87]. Midostaurin is now being investigated in combination with chemotherapy in children with newly diagnosed, FLT3-mutated AML (NCT03591510), which is ongoing.

Second-generation FLT3 inhibitors are much more highly selective in targeting FLT3, thereby showing more limited off-target toxicity [6]. Quizartinib has completed investigation through a Phase I clinical trial in combination with salvage chemotherapy in r/r childhood leukemia, both harboring FLT3 wild-type and FLT3-ITD mutations, and demonstrated 4/17 CR and 10/17 stable disease (SD). Of the 7 FLT3-ITD mutated patients on the trial, there were 3 CR and 4 SD, demonstrating some clinical efficacy in all FLT3-mutated patients [88]. Quizartinib is currently being investigated in a Phase I/II clinical trial in combination with re-induction chemotherapy and as monotherapy for maintenance in r/r, FLT3-ITD mutated AML in children (NCT03793478).

Finally, gilteritinib is another potent and highly selective second generation FLT3 inhibitor that exerts dual activity against FLT3 and AXL, an additional receptor tyrosine kinase that promotes proliferation and survival of AML cells [6]. Due to promising results from a Phase I/II trial on giltertinib in adults with r/r AML harboring both wild-type and FLT3 mutations [89], a randomized Phase III trial was conducted comparing gilteritinib monotherapy versus salvage chemotherapy in adults with r/r, FLT3-mutated AML. Patients who received gilteritinib demonstrated a significantly higher CR/Cri rate (34% vs. 15%), longer median OS (9.3 months vs. 5.6 months), and longer post-transplant survival (16.2 months vs. 8.4 months) as compared to those who received salvage chemotherapy, resulting in FDA approval for adults with r/r, FLT3-mutated AML [90]. Gilteritinib is planned to be investigated in the upcoming COG AML trial for children with newly diagnosed AML (AAML1831).

## 5. Conclusions

In summary, the last several years have been pivotal for AML clinicians, researchers, and patients, representing a period of robust discovery and development of targeted drugs for AML. Many of these drugs, as described in this review, have advanced from bench to bedside and are being evaluated in adults with either ongoing or emerging studies in children. The impact of these ADCs, small molecule inhibitors, and FLT3 inhibitors on improving survival in children with leukemia and other cancers remains to be seen as pediatric clinical trials become increasingly available. While the evaluation of these novel drugs as monotherapy is a critical first step, it will be equally critical to test their safety and efficacy in strategic combination with cytotoxic chemotherapy and/or other novel therapies. The standard of care for upfront AML therapy in children remains a risky, stratified, multi-agent chemotherapy regimen, but as evidenced by the last several landmark COG AML trials, incorporation of targeted therapies, including Gemtuzumab ozogamicin and tyrosine kinase inhibitors targeting FLT3-ITD mutations, are improving outcomes. The AML molecular landscape has evolved along with our understanding of the biology and genetic makeup of AML, and the potential of ongoing research may dramatically inform future treatment options.

## Figures and Tables

**Table 1:** Selected novel AML therapies currently in clinical trials in children and adults.

Therapy	Type	Target	Clinical Trial	Patient Population
Gemtuzumab ozogamicin + chemotherapy	Antibody drug conjugate	CD33	Phase III COG AAML1831 GO included in standard of care treatment backbone—in development	Newly diagnosed AML in children
Anetumab ravtensine	Antibody drug conjugate	Mesothelin	Phase I (COG AAML2011)—in development	≥2nd relapse AML in children with mesothelin+ AML
IMGN632	Antibody drug conjugate	CD123	Phase I (NCT03386513) Phase I/II—in development	R/R AML Adults with CD123+ AML and other hematologic malignancies R/R AML in children
Uproleselan (GMI-1271)	Small molecule inhibitor	E-selectin	Randomized phase III (NCT03616470)	R/R AML in adults
Pinometostat + Azacitidine	Small molecular inhibitor	DOT1L	Phase I/II (NCT03701295)	Newly diagnosed or R/R AML with KMT2A rearrangement in adults
SNDX-5613 (VTP-50469)	Small molecule inhibitor	KMT2A rearrangement or NPM1 mutation	Phase I/II (NCT04065399)	Phase I: R/R acute leukemia Phase II in adults: Cohort 2A: MLLr ALL/MPAL Cohort 2B: MLLr AML Cohort 2C: NPM1c AML
KO = 539	Small Molecule Inhibitor	KMT2A rearrangement	Phase I/II (NCT04067336)	R/R AML in adults
Trametinib	Small molecule inhibitor	RAS-pathway mutations	COG Phase II ADVL1521 (NCT03190915)	R/R juvenile myelomonocytic leukemia (JMML) in children
Idasanutlin+Cytarabine	Small molecule inhibitor	MDM2 antagonist	Randomized Phase III (NCT02545283)	R/R AML with WT and mutated TP53 in adults
ALRN-6924 (dual MDM2/MDMX inhibitor)	Small molecule inhibitor	MDM2 antagonist	Phase I (NCT03654716)	R/R AML, ALL, MPAL, or other undifferentiated acute leukemia (Cohort C) with WT TP53 in children
APR-246	Small molecule inhibitor	TP53	Randomized Phase III (NCT03745716)	TP53-mutated MDS in adults
Sorafenib + Palbociclib	Tyrosine kinase inhibitor	FLT3	Phase I (NCT03132454)	R/R AML and ALL in adolescents and adults
Midostaurin + chemotherapy	Tyrosine kinase inhibitor	FLT3	Phase II (NCT03591510)	Newly diangosed FLT3-mutated AML in children
Quizartinib + chemotherapy	Tyrosine kinase inhibitor	FLT3	Phase I/II (NCT03793478)	R/R FLT3-mutated AML in children
Gilteritinib + chemotherapy	Tyrosine kinase inhibitor	FLT3	Phase III (COG AAML1831)—in development	Newly diagnosed FLT3-mutated AML in children
